# *Subtercola vilae* sp. nov., a novel actinobacterium from an extremely high-altitude cold volcano lake in Chile

**DOI:** 10.1007/s10482-017-0994-4

**Published:** 2017-12-06

**Authors:** Alvaro S. Villalobos, Jutta Wiese, Pablo Aguilar, Cristina Dorador, Johannes F. Imhoff

**Affiliations:** 1 Marine Microbiology, GEOMAR Helmholtz Centre for Ocean Research Kiel, Düsternbrooker Weg 20, 24105 Kiel, Germany; 2 Laboratorio de Complejidad Microbiana y Ecología Funcional and Departamento de Biotecnología, Facultad de Ciencias del Mar y Recursos Biológicos, Universidad de Antofagasta, Antofagasta, Chile; 3 Lake and Glacier Ecology Research Group, Institute of Ecology, University of Innsbruck, Techniker Str. 25, 6020 Innsbruck, Austria; 4 Centre for Biotechnology and Bioengineering (CeBiB), Universidad de Antofagasta, Antofagasta, Chile

**Keywords:** Cold environments, Llullaillaco volcano, Microbacteriaceae, New species, *Subtercola vilae*

## Abstract

A novel actinobacterium, strain DB165^T^, was isolated from cold waters of Llullaillaco Volcano Lake (6170 m asl) in Chile. Phylogenetic analysis based on 16S rRNA gene sequences identified strain DB165^T^ as belonging to the genus *Subtercola* in the family *Microbacteriaceae*, sharing 97.4% of sequence similarity with *Subtercola frigoramans* DSM 13057^T^, 96.7% with *Subtercola lobariae* DSM 103962^T^, and 96.1% with *Subtercola boreus* DSM 13056^T^. The cells were observed to be Gram-positive, form rods with irregular morphology, and to grow best at 10–15 °C, pH 7 and in the absence of NaCl. The cross-linkage between the amino acids in its peptidoglycan is type B2γ; 2,4-diaminobutyric acid is the diagnostic diamino acid; the major respiratory quinones are MK-9 and MK-10; and the polar lipids consist of phosphatidylglycerol, diphosphatidylglycerol, 5 glycolipids, 2 phospholipids and 5 additional polar lipids. The fatty acid profile of DB165^T^ (5% >) contains iso-C14:0, iso-C16:0, anteiso-C15:0, anteiso-C17:0, and the dimethylacetal iso-C16:0 DMA. The genomic DNA G+C content of strain DB165^T^ was determined to be 65 mol%. Based on the phylogenetic, phenotypic, and chemotaxonomic analyses presented in this study, strain DB165^T^ (= DSM 105013^T^ = JCM 32044^T^) represents a new species in the genus *Subtercola*, for which the name *Subtercola vilae* sp. nov. is proposed.

## Introduction

Members of the family *Microbacteriaceae* are widely distributed in terrestrial and aquatic environments or associated with macroorganisms (Evtushenko 2012). Some representatives, including species of the genus *Subtercola*, have been found in cold environments such as glacial ice (Christner et al. 2007), boreal groundwater (Männistö et al. 2000), and Antarctic sediments (Li et al. 2010). At present the genus *Subtercola* contains three validly named species, *Subtercola boreus*, *Subtercola frigoramans* and *Subtercola lobariae*; the first two were isolated from Finnish groundwater (Männistö et al. 2000) and the third from the lichen *Lobaria retigera* (Si et al. 2017). Based on the high similarity of 16S rRNA gene sequences (> 96%), other isolates from cold habitats, such as Antarctic and Artic waters as well as glaciers were found to be affiliated to *Subtercola* (Singh et al. 2014; Zhang et al. 2013; Peeters et al. 2011).

In this study, we characterise strain DB165^T^, isolated from a water sample of Llullaillaco Volcano Lake (6170 m) in Chile, one of the highest-elevation lakes on Earth. According to its distinct properties, strain DB165^T^ is proposed as the type strain of the new species *Subtercola vilae*.

## Materials and methods

### Isolation and cell morphology

Strain DB165^T^ was obtained from a water sample collected at the Llullaillaco volcano lake (S24°42.878′, W68°33.310′) on 18 January 2013, using R2A medium (DIFCO) supplemented with 18 g agar l^−1^. Pure cultures were obtained after three successive transfers of single colonies to R2A medium plates. Stock cultures were maintained in SGG medium containing 10 g starch, 10 g glucose, 10 ml glycerol (99.7% v/v), 5 g soy peptone, 2.5 g corn steep solids, 2 g yeast extract, 3 g CaCO_3_, 1 g NaCl, and 18 g agar in 1 l deionised water (Goodfellow and Fiedler 2010). DB165^T^ was cryopreserved using CRYOBANK (Mast Diagnostica GmbH, Germany) for long term storage at − 80 °C.

Gram-staining was prepared using the Color Gram 2 kit (BioMérieux, France), following the manufacturer’s protocol. Endospore staining was performed using the green malaquite method and light microscopy (Schaeffer and Fulton 1933). Cell morphology, shape and size were determined using scanning electron microscopy (SEM) according to Gärtner et al. (2008), after cultivation of trypticase soy medium (Trypticase Soy Broth (Becton, Dickinson and company, France) supplemented with 18 g agar l^−1^.

The reference strains *S. boreus* DSM 13057^T^ and *S. frigoramans* DSM 13057^T^ were obtained from the German Collection of Microorganisms and Cell Cultures (DSMZ) and cultured under the same conditions as strain DB165^T^ for comparative purposes.

### Physiological characteristics

Enzyme activities and utilisation of carbon sources for strain DB165^T^, *S. boreus* DSM 13057^T^ and *S. frigoramans* DSM 13057^T^ were examined using API ZYM, API 20E and API 50CH (BioMérieux, France), following the manufacturer’s recommendations. The effect of sodium chloride (0, 0.1, 0.3, 0.6, 0.9, 1, 2.5, 5, 7.5, and 10% w/v) and pH (2, 3, 4, 5, 6, 7, 8, 9 and 10) on the growth was tested according to Kutzner (1981), using ISP2 medium containing 4 g yeast extract, 10 g malt extract, 4 g dextrose, and 18 g agar in 1 l of distilled water. The optimal range of temperature was tested at 5, 10, 15, 20, 28 and 30 °C using SGG medium.

### Chemotaxonomic analyses

Polar lipids were extracted according to a modified protocol of Bligh and Dyer (1959), and the total lipid material was detected using molybdatophosphoric acid and specific functional groups were detected using spray reagents specific for defined functional groups (Tindall et al. 2007). The lipoquinones were extracted and identified using the two-stage method described by Tindall (1990a, b). After cultivation at 25 °C, fatty acid methyl esters were obtained by saponification, methylation and extraction using minor modifications of the method of Miller (1982) and Kuykendall et al. (1988) The fatty acid methyl esters mixtures were separated and identified using the Sherlock Microbial Identification System (MIS) (MIDI, Microbial ID, Newark, DE 19711, USA).

The peptidoglycan was obtained from 4 g wet weight cell pellet according to the method of Schleifer (1985). The peptidoglycan analyses were performed according to Schumann (2011).

Analyses of polar lipids, respiratory quinones, whole-cell fatty acids and peptidoglycan analyses were carried out by the Identification Service of the DSMZ—Deutsche Sammlung von Mikroorganismen und Zellkulturen GmbH (Braunschweig, Germany).

### DNA base composition

DNA was extracted using the DNeasy Blood & Tissue kit (QIAGEN). The G+C content was calculated from the genome sequence, which was determined with Nextseq 500 (Illumina). The quality of the sequences was checked and filtrated using Trimmomatic (adapters, > Q30, > 1000 bp) (Bolger et al. 2014). The genome was assembled using SPAdes (Kmer = 121) (Bankevich et al. 2012).

### Phylogenetic analyses

DNA was extracted using the DNeasy Blood & Tissue kit (QIAGEN) with modifications. The 16S rRNA gene sequence was amplified by PCR using PureTaq Ready-To-Go PCR beads (GE Healthcare) and sequencing according to Gärtner et al. (2008).

The 16S rRNA gene sequence of strain DB165^T^ was aligned with sequences of 22 selected type strains of the family *Microbacteriaceae*, including members of the genera *Subtercola*, *Frondihabitans* and *Agreia*, and in addition *Cellulomonas carbonis* KCTC 19824^T^ as outgroup using SINA (Pruesse et al. 2012). Phylogenetic trees were constructed using the neighbour-joining (Saitou and Nei 1987) and maximum-likelihood algorithms using MEGA version 6.0 (Tamura et al. 2013). The tree topologies were evaluated with bootstrap analyses based on 1000 replicates.

## Results

### Morphological and physiological characteristics

**Fig. 1:**
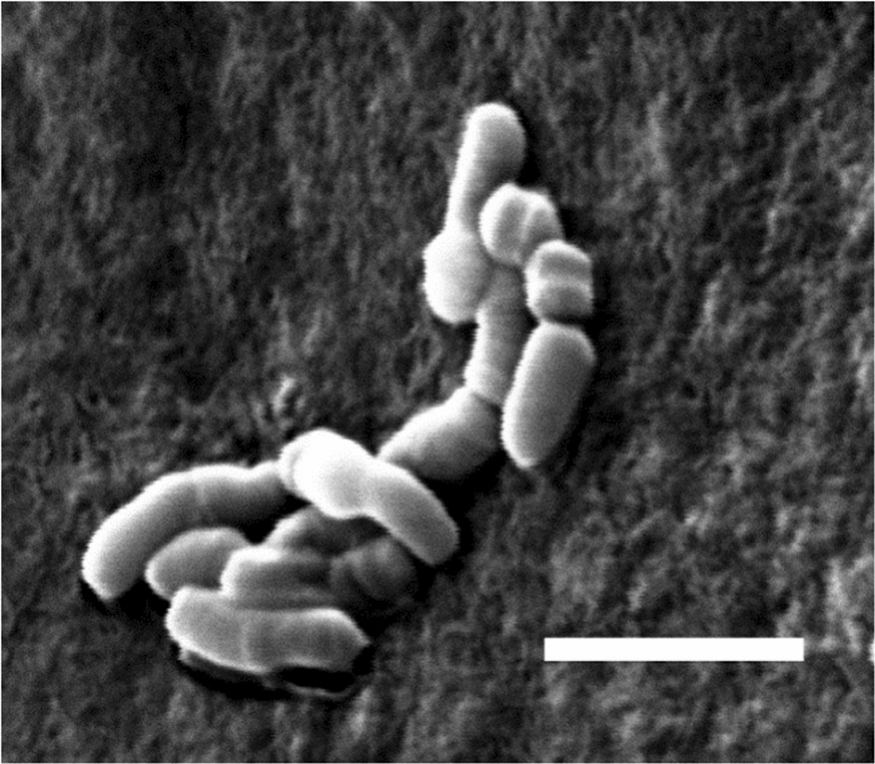
Scanning electron micrograph of strain DB165^T^ grown on trypticase soy medium for 7 days at 28 °C. Scale bar indicates 2 µm

Colonies of strain DB165^T^ were observed to be sticky, golden yellow after growth at the optimal growth temperature of 10–15 °C for 6–7 days (also after 7–10 days at 28 °C), but are pale yellow after growth at 5 °C for 2–3 weeks. Optimum growth is observed at 10–15 °C (range from 5 to 28 °C). No growth occurs at 30 °C. Strain DB165^T^ tolerates only low concentrations (up to 0.9%) of NaCl and grows best in the absence of NaCl. The pH range for growth is from 5 to 8, with an optimum at pH 7. Cells show no motility and form no spores, they are irregular short rods of 0.5 µm width and 1.0–1.2 µm length, Gram-positive and have an irregular shape as seen under SEM. Some of the cells are thicker at the ends. Occasionally, coccoid cells were observed (Fig. 1). Variable cell shapes have also been reported for *S. boreus* and *S. frigoramans* (Männistö et al. 2000).

The metabolic properties of strain DB165^T^, in comparison with the type strains of *S. boreus* and *S. frigoramans* are shown in Table 1. Strain DB165^T^ was found to metabolise inositol, d-sorbitol, d-sucrose, d-melibiose, glycerol, l-arabinose, d-xylose, methyl-β-d-xylopyranoside, d-galactose, d-glucose, d-fructose, d-mannose, l-rhamnose, d-mannitol, amygdalin, arbutin, esculin, salicin, d-cellobiose, d-maltose, d-trehalose, d-melezitose, gentiobiose and d-turanose.

**Table 1:** Physiological characteristics of strain DB165^T^ compared to the type strains of *S. frigoramans* and *S. boreus*

	*S. vilae*	*S. frigoramans*	*S. boreus*
	DB165^T^	DSM 13057^T^	DSM 13056^T^
APIZYM			
Alkaline phosphatase	−	(+)	+
Esterase (C4)	+	(+)	+
Valine arylamidase	(+)	(+)	+
Cysteine arylamidase	(+)	−	+
Trypsin	(+)	−	−
α-Galactosidase	(+)	−	−
β-Glucoronidase	−	−	+
*n*-Acetyl-β-glucosaminidase	−	(+)	−
α-Mannosidase	−	(+)	(+)
API20E			
Arginine dihydrolase	−	(+)	−
Citrate as unique carbon source	−	(+)	(+)
Tryptophan deaminase	+	−	−
Tryptophanase	+	−	−
Voges–Proskauer test (production of acetoin)	+	−	−
API 50CH			
d-Arabinose	−	−	+
d-Ribose	−	−	+
Methyl-β-d-xylopyranoside	+	−	−
d-Mannose	+	−	+
Arbutin	+	−	−
Salicin	+	−	−
d-Lactose (bovine origin)	−	−	+
d-Melezitose	+	−	−
Gentiobiose	+	−	−

In the present study, all *Subtercola* strains showed positive activity in the tests for esterase lipase (C8), leucine arylamidase, acid phosphatase, Naphthol-AS-Bl-phosphohydrolase, α-glucosidase, β-glucosidase, β-galactosidase (weak in API ZYM test), d-glucose, d-mannitol, inositol, d-sorbitol, l-rhamnose, d-sucrose, d-melibiose, amygdalin, l-arabinose, glycerol, d-xylose, d-galactose, d-fructose, esculin, d-cellobiose, d-maltose, d-trehalose and d-turanose; and negative activity for lipase (C14), α-chymotrypsin, α-fucosidase, lysine decarboxylase, ornithine decarboxylase, sulfide production, urease, gelatinase, erythritol, l-xylose, l-sorbose, dulcitol, methyl-α-d-mannopyranoside, methyl-α-d-glucopyranoside, *N*-acetylglucosamine, inulin, d-raffinose, starch, glycogen, xylitol, d-lyxose, d-tagatose, d-fucose, d-arabitol, l-arabitol, potassium gluconate, potassium 2-ketogluconate and potassium 5-ketogluconate

+ positive activity, (+) weak activity, − no activity

### Chemotaxonomic characteristics

The polar lipids of the strain DB165^T^ were found to consist of phosphatidylglycerol, diphosphatidylglycerol, 5 unidentified glycolipids, 2 unidentified phospholipids and unidentified 5 lipids. The diamino acid in the peptidoglycan was identified as 2,4-diaminobutyric acid (DAB). The molar ratio of alanine:glycine:glutamic acid:DAB was 1.1:1.0:0.04:1.7. Instead of glutamic acid, high amounts of 3-hydroxyl-glutamic acid were found. Assuming that much of the glutamic acid is replaced by 3-hydroxyl-glutamic acid, and despite this replacement, the amino acid composition is consistent with peptidoglycan type B2γ. The major isoprenoid quinones of strain DB165^T^ were identified as MK-9 (47%) and MK-10 (39%). Minor amounts of MK-11 (6%) and MK-8 (4%) were also found to be present. The G+C content of the genomic DNA of the strain DB165^T^ was determined to be 65.0 mol%.

The major fatty acids of the strain DB165^T^ were identified as iso- and anteiso-saturated C15 and C16 fatty acids with anteiso-C15:0 (50%), iso-C16:0 (17%) and iso-C16:0 DMA (17%) as major components (Table 2).

**Table 2:** Fatty acid profiles of strain DB165^T^, *S. frigoramans* DSM 13057^T^, *S. boreus* DSM 13056^T^ and *S. lobariae* DSM 103962^T^

	*S. vilae*	*S. frigoramans*	*S. boreus*	*S. lobariae*
	DB165^T^	DSM 13057^T^	DSM 13056^T^	DSM 103962^T^
14:0	Tr	–	–	–
14:0 2-OH	–	–	–	10.3
16:0	Tr	–	–	Tr
i-14:0	5.5	6.7	Tr	2.3
i-15:0	Tr	Tr	4.3	1.4
i-16:0	17.2	10.2	4.2	6.7
i-17:0	–	–	–	–
a-15:0	50.0	46.1	51.6	68.8
a-17:0	6.7	6.8	3.5	4.2
a-15:1	–	–	Tr	–
16:0 DMA	Tr	–	Tr	–
i-15:0 DMA	Tr	–	1.7	1.5
i-16:0 DMA	17.0	13.3	11.9	6.9
a-15:0 DMA	3.2	10.3	11.0	9.7
a-17:0 DMA	1.9	2.9	4.0	2.6
References	This study	Männistö et al. (2000)	Männistö et al. (2000)	Si et al. (2017)

Percent of total peak area of ion chromatograms is indicated

Cells for fatty acid and dimethyl acetals were grown at 25 °C, except for *S. lobariae* where DMAs were obtained from cells cultivated at 20 °C

*Tr* traces (< 1%), − not detected, *a-* anteiso-branched fatty acid, *i-* iso-branched fatty acid, *DMA* 1,2 dimethyl acetals

### 16S rRNA gene sequence analyses

**Fig. 2:**
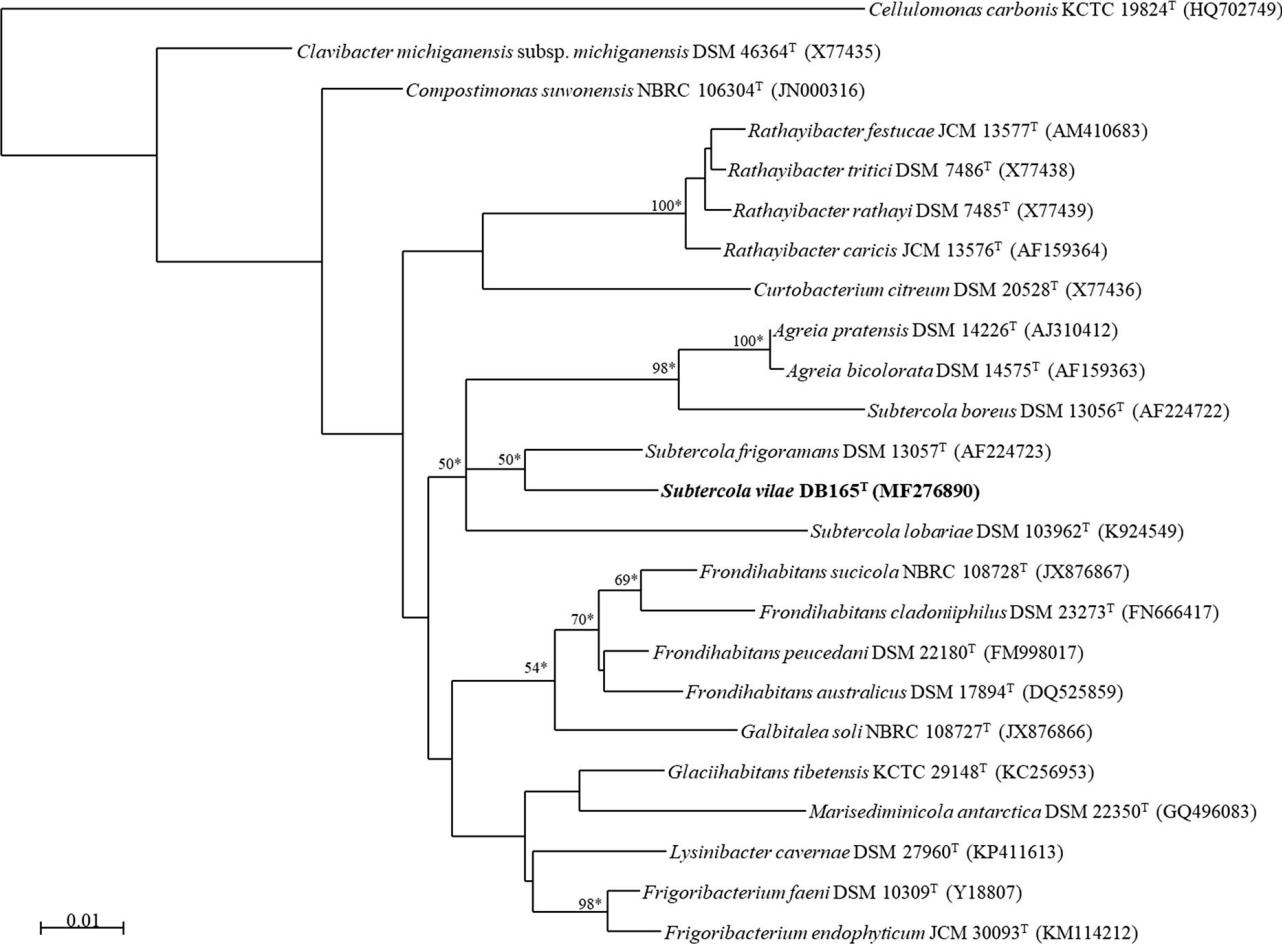
Maximum-likelihood phylogenetic tree based on 16S rRNA gene sequence comparison of strain DB165^T^ and species of the family *Microbacteriaceae* with *Cellulomonas carbonis* KCTC 19824^T^ as outgroup. Numbers at the nodes represent bootstrap support (%) based on the analysis of 1000 bootstrap replications, asterisks indicate branches of the tree that were also recovered using neighbour joining algorithm. Only bootstrap values ≥ 50% are indicated. Genbank accession numbers are given in parentheses. Bar indicates 0.01 substitutions per site

The 16S rRNA gene sequence of strain DB165^T^ (1432 bp, deposited under Genbank accession number MF276890) matches to different *Subtercola* species in a range of 96.1–97.4% of similarity, i.e., *S. frigoramans* DSM 13057^T^ (97.4% similarity), *S. lobariae* DSM 103962^T^ (96.7% similarity) and *S. boreus* DSM 13056^T^ (96.1% similarity). However, it also showed high similarity to *Frondihabitans* species, including *Frondihabitans peucedani* DSM 22180^T^ (96.8% similarity) and *Frondihabitans australicus* DSM 17894^T^ (96.6% similarity). The phylogenetic analysis (Fig. 2) based on the consensus 16S rRNA gene sequences (1416 bp) showed that strain DB165^T^ forms a cluster with *Subtercola* species and *Agreia* with a strong bootstrap support, while *Frondihabitans* species cluster together, but the separation with the *Subtercola*/*Agreia* cluster is not well supported by bootstrap analysis. In the *Subtercola* clade, strain DB165^T^ is found in a distinct cluster with *S. frigoramans* DSM 13057^T^ with strong bootstrap support.

### Genome characteristics

The genome of strain DB165^T^ has a size of approx. 4 Mb and give some hints on the strains’ potential to adapt to the harsh conditions that are found at Llullaillaco volcano lake. These include mechanisms concerning membrane fluidity, biosynthesis of cryoprotectants, and ice-interacting proteins (data not shown).

## Discussion

*Subtercola* species are characterised by a peptidoglycan type B2γ with DAB as diamino acid, MK-9 and MK-10 as major respiratory quinones, and a similar polar lipids profile (Table 3).

**Table 3:** Diagnostic key characteristics of members of the genera *Subtercola*, *Agreia,* and *Frondihabitans* in comparison to strain DB165^T^

	*Subtercola vilae* DB165^T^	*Subtercola*	*Agreia*	*Frondihabitans*
Peptidoglycan type	B2γ	B2γ	B2γ	B2β
Cell wall diamino acid	DAB	DAB	l-DAB	d-Orn
			d-Orn	
Respiratory quinones	MK-9, MK-10	MK-9, MK-10	MK-10, MK-11	MK-7, MK-8, MK-9
Polar lipids	PG, DPG, GL, PL, L	PG, DPG, GL, PL	PG, DPG	PG, DPG, GL, AL, PL
Major cellular fatty acids (> 10%)	a-15:0, i-16:0	a-15:0, i-16:0	a-15:0, i:16:0, a-17:0	18:1, 14:0 2-OH, a-15:0
Major 1,1-dimethyl acetals (> 5%)	i-16:0 DMA	i-16:0 DMA, a-15:0 DMA	ND	ND
G+C content (mol%)	65	64–68	65–67	65–71
Isolation source	Volcano lake at 6170 m asl	Boreal groundwater, lichen	Leaf gall, phyllosphere of grasses	Associated to plants and lichen
References	This work	Männistö et al. (2000), Si et al. (2017)	Evtushenko et al. (2001), Behrendt et al. (2002)	Kim et al. (2014)

*PG* phosphatidylglycerol, *DPG* diphosphatidylglycerol, *GL* glycolipids, *PL* phospholipids, *AL* aminolipid, *L* lipids, *ND* not detected

Strain DB165^T^ has a peptidoglycan type B2γ in which the glutamic acid is almost completely replaced by 3-hydroxyl-glutamic acid, as is found in other described *Subtercola* species (Männistö et al. 2000; Si et al. 2017). The metabolic characteristics that differentiate strain DB165^T^ from *S. boreus* and *S. frigoramans* are utilisation of methyl-β-d-xylopyranoside, arbutin salicin, d-melezitose and gentobiose as carbon sources and the enzymatic activities of trypsin, α-galactosidase, tryptophanase deaminase and tryptophanase, as well as the production of acetoin. In contrast to strain DB165^T^, *S. frigoramans* exhibited as unique features arginine dihydrolase and *n*-acetyl-β-glucosaminidase. *S. frigoramans* and *S. boreus* can use citrate as unique carbon source and showed the enzymatic activities of alkaline phosphatase and α-mannosidase. *S. boreus* showed β-glucoronidase enzymatic activity and can use d-arabinose, d-ribose, and d-lactose, carbon sources that strain DB165^T^ and *S. frigoramans* cannot use. The fatty acid profile of strain DB165^T^ is similar to those of the type strains of other *Subtercola* species. Major differences were observed in the content of iso-C16:0, with 17% in strain DB165^T^ compared to 4.2–10.2% in the other *Subtercola* species and a low content of 3% anteiso-C15:0 DMA, compared to 9.7–11.0% in the other *Subtercola* species (Table 2).

The phylogenetic analysis of the 16S rDNA gene sequence clearly shows the close relationship of strain DB165^T^ to *Subtercola* species rather than to *Agreia* and *Frondihabitans* species. The genus *Agreia* forms a sub-cluster within *S. boreus* (this study and Si et al. 2017) and different chemotaxonomic traits have been proposed to distinguish the two genera (Schumann et al. 2003). Though both genera have a cross-linkage between the amino acids in the peptidoglycan of type B2γ, in the case of *Subtercola* species, the cross-linkages have DAB, while *Agreia* species have l-DAB connected to d-Orn. *Frondihabitans* species can be clearly distinguished by their peptidoglycan, which is of the B2β type (Zhang et al. 2007). Fatty acids play an important role in the differentiation of the genera. The presence of 1,2 dimethyl acetals (iso-C16:0 DMA and anteiso-C17:0 DMA) is observed in all *Subtercola* species, while *Agreia* only contains iso-C15:0 DMA in low proportions (≤ 4%) (Schumann et al. 2003; Behrendt et al. 2002). *Frondihabitans* species have a fatty acid profile very distinct from those of *Subtercola* and *Agreia* species, having C18:1 and C14:0 2-OH as major fatty acids but lacking 1,2 dimethyl acetals. The major menaquinones of *Subtercola* species, including the strain DB165^T^, comprise MK-9 and MK-10, while in *Agreia bicolorata* DSM 14575^T^ MK-10 and in *Agreia pratensis* DSM 4246^T^ MK-10 and MK-11 are dominant, and in *Frondihabitans* spp. MK-8 and MK-7. The presence of MK-9 as a major component can be used as a marker to differentiate these three genera (Table 2). It should be mentioned that *A. pratensis*, which was originally classified as *Subtercola pratensis*, contains MK-10 (51%) and MK-11 (21%) as major menaquinones, but in addition 13% of MK-9 (Behrendt et al. 2002, Evtushenko et al. 2001). Irrespective of the problematic taxonomic position of *Agreia* species and the similarity of 16S rRNA gene sequences with *Frondihabitans*, the phylogenetic relationships (Fig. 2) and chemotaxonomic criteria clearly support the classification of strain DB165^T^ as a member of the genus *Subtercola* (Table 3). Based on the phenotypic and genetic analyses presented in this work, strain DB165^T^ is considered to represent a new species of the genus *Subtercola*, for which the name *Subtercola vilae* sp. nov. is proposed. The Digital Protologue database (Rosselló-Móra et al. 2017) TaxoNumber for strain DB165^T^ is TA00217.

## Description of *Subtercola vilae* sp. nov.

*Subtercola vilae* (vi’lae, of Vila, named in honour of Irma Vila, a Chilean limnologist with outstanding contributions to the microbiology and ecology of lakes in the Chilean Altiplano and Atacama Desert).

Cells are short, irregular rods 0.5 µm wide and 1.0–1.2 µm long. Colonies are golden yellow, circular convex. Growth occurs chemoheterotrophically under oxic conditions. Optimum growth is at 10–15 °C (range from 5 to 28 °C), at pH 7 (range from pH 5 to 8) and in the absence of NaCl. Cells produce esterase C4, esterase lipase C8, leucine arylamidase, acid phosphatase, naphthol-AS-BI-phosphohydrolase, α- and β-glucosidase, tryptophan deaminase, tryptophanase and acetoin. Weak activity is observed for valine arylamidase, cysteine arylamidase, trypsin, and α- and β-galactosidase. Carbon sources used under oxic conditions include inositol, d-sorbitol, d-sucrose, d-melibiose, glycerol, l-arabinose, d-xylose, methyl-β-d-xylopyranoside, d-galactose, d-glucose, d-fructose, d-mannose, l-rhamnose, d-mannitol, amygdalin, arbutin, esculin, salicin, d-cellobiose, d-maltose, d-sucrose, d-trehalose, d-melezitose, gentiobiose and d-turanose. The cell-wall peptidoglycan is type B2γ with DAB as the diagnostic amino acid and 3-hydroxyl-glutamic acid instead of glutamic acid. Major menaquinones are MK-9 and MK-10. Polar lipids comprise phosphatidylglycerol, diphosphatidylglycerol, 5 unidentified glycolipids, 2 unidentified phospholipids and 5 unidentified lipids. The major cellular fatty acids are anteiso-C15:0, iso-C16:0, anteiso-C17:0, and iso-C14:0, while C14:0 and C16:0 are found only in traces. Major dimethylacetals are iso-C16:0 DMA, anteiso-C15:0 DMA and anteiso-C17:0 DMA, while C16:0 DMA and iso-C15:0 DMA are present in trace amounts. The G + C content of the DNA of the type strain is 65.0 mol%.

The type strain DB165^T^ (= DSM 105013^T^ = JCM 32044^T^) was isolated from Llullaillaco volcano lake in Chile. The GenBank/EMBL/DBBJ accession number for the 16S rRNA of strain DB165^T^ is MF276890.
